# Elderly: Coping with sleep disorders

**DOI:** 10.1192/j.eurpsy.2021.1484

**Published:** 2021-08-13

**Authors:** D. Silva, R. Martins, F. Polido, M.D.C. Cruz

**Affiliations:** Psychiatry, Centro Hospitalar Universitário do Algarve, Portimão, Portugal

**Keywords:** Elderly, sleep

## Abstract

**Introduction:**

Sleep problems are a common presenting symptom of elderly patients to Primary care physicians and Psychiatrists. Almost half of seniors over age 65 who live at home are dissatisfied with their sleep, and nearly two-thirds of those residing in nursing home facilities suffer from sleep disorders. Chronic and pervasive sleep complaints and disturbances are frequently associated with excessive daytime sleepiness and may result in impaired cognition, diminished intellect, poor memory, confusion, and psychomotor retardation.

**Objectives:**

The aim of this article is to sumarize and explore the facts envolving sleep disorders, discusses approaches to treatment and highlights new research in the area of geriatric sleep disorders.

**Methods:**

An online bibliographic search was carried out on PubMed and Medline using the keywords “Elderly”, “sleep” and “Psychiatry”.

**Results:**

Management of sleep disorders is complicated by the risk of side effects of pharmacologic treatment approaches, and thus nonpharmacologic strategies are preferred when possible. Additionally, many of the pharmacologic strategies used in treating younger adults have not been studied adequately in the geriatric population, and more specifically in patients with underlying cognitive disorders, making treatment choices difficult.

**Conclusions:**

This review has provided insights into the biopsychosocial impact of sleep disorders in the elderly, as this group pose unique challenges for diagnosis and treatment. Sleep changes in the elderly may have a far broader impact on geriatric health than originally thought, with implications for AD and delirium, and further research is needed in these areas as well.

